# Sensitive methods for the detection of an insertion in exon 20 of the *HER2* gene in the metastasis of non-small cell lung cancer to the central nervous system

**DOI:** 10.3892/ol.2013.1495

**Published:** 2013-07-26

**Authors:** PAWEŁ KRAWCZYK, MARCIN NICOŒ, TOMASZ POWRÓZEK, RADOSŁAW MLAK, MAREK SAWICKI, BOŻENA JAROSZ, BEATA PAJĄK, KRZYSZTOF KUCHARCZYK, DARIUSZ STENCEL, TOMASZ TROJANOWSKI, JANUSZ MILANOWSKI

**Affiliations:** 1 Department of Pneumonology, Oncology and Allergology, Medical University of Lublin, Lubin 20-954, Poland; 2 Postgraduate School of Molecular Medicine, Warsaw 02-091, Poland; 3 Department of Thoracic Surgery, Medical University of Lublin, Lubin 20-954, Poland; 4 Department of Neurosurgery and Pediatric Neurosurgery, Medical University of Lublin, Lubin 20-954, Poland; 5 BioVectis Ltd., Kucharczyk TE, Warsaw 02-106, Poland; 6 Electron Microscopy Platform, Mossakowski Medical Research Centre, Polish Academy of Sciences, Warsaw 02-106, Poland; 7 Boehringer Ingelheim Poland, Warsaw 02-675, Poland; 8 Institute of Agricultural Medicine, Lublin 20-090, Poland

**Keywords:** non-small cell lung cancer, metastasis to the central nervous system, HER2 mutation

## Abstract

The HER2 (ErbB2/neu) protein is a member of the HER (ErbB) receptor family (EGFR, HER2, HER3 and HER4) that expresses tyrosine kinase activity in the intracellular domain. EGFR and HER2 overexpression is observed in numerous types of cancer, nevertheless, the susceptibility of patients with non-small cell lung cancer (NSCLC) to therapy with EGFR and HER2 tyrosine kinase inhibitors (TKIs) depends on mutations present in the respective coding genes (driver mutations). In the present study, PCR and amplified DNA fragment length analysis (FLA) were used along with the multi-temperature single-strand conformation polymorphism (MSSCP) technique in order to identify the 12 base pair insertion in exon 20 of the *HER2* gene in 143 patients with NSCLC metastasis to the central nervous system. The prevalence of the *HER2* gene mutation was correlated with mutations in the *EGFR* and *BRAF* genes. The insertion in exon 20 of the *HER2* gene was observed in a single 77-year-old, non-smoking male, with poorly-differentiated adenocarcinoma of the lung (1.5% of adenocarcinoma patients). No other genetic abnormalities were identified in this patient. In the therapy of NSCLC patients with *HER2* gene mutations, drugs that inhibit the EGFR and HER2 receptors, for example afatinib, may be effective. The identification of other driving mutations in NSCLC cells appears to be key to the appropriate qualification of molecular targeted therapies.

## Introduction

The HER (ErbB) receptor family consists of four receptors: HER1 (EGFR, ErbB1), HER2 (Neu, ErbB2), HER3 (ErbB3) and HER4 (ErbB4) that express tyrosine kinase activity in the intracellular domain. The ErbB abbreviation derives from the erythroblastic leukemia oncogenic virus (erythroblastic leukemia viral oncogene), which is structurally homologous to human HER receptors (1). The signal for epithelial cell proliferation is transduced to the cell nucleus as a result of the homo- and heterodimerization of HER receptors and activation of the complex by corresponding ligand binding. The preferred partner for EGFR heterodimerization is the HER2 receptor, which is also known to be overexpressed in breast cancer (2). Mutations within the tyrosine kinase domain of the *EGFR* gene, mainly deletions in exon 19 or substitution L858R in exon 21, are detected in ~10% of Caucasian patients with non-small cell lung cancer (NSCLC) (3). Additionally, the *EGFR* gene mutation affects the efficacy of *EGFR* tyrosine kinase inhibitors (TKIs). There is certain evidence that *HER2* gene mutations, and perhaps the high expression of the *HER2* receptor, may be involved in the etiology of certain NSCLC cases (4).

Mutations in the *HER2* gene tyrosine kinase domain are extremely rare in NSCLC patients (5). Preliminary data show that the prevalence is not higher than 2% in the general patient population (6). The mutations are most often indicated in non-smoking females with adenocarcinoma of the lung (7). The most significant mutations are two different insertions of 12 base pairs, which impair the reading frame in exon 20 of the *HER2* gene: A775YVMA (66% of all detected mutations in the *HER2* gene) or M774AYVM. These mutations are identical to the insertion of nine base pairs in exon 20 of the *EGFR* gene, which makes the structure of the tyrosine kinase domain of the HER2 protein similar to the structure of the tyrosine kinase domain of the *EGFR* gene, modified by the mentioned mutations (5). Based on this, it is assumed that A775YVMA or M774AYVM mutations of the *HER2* gene cause similar consequences to the mutations in exon 20 of the *EGFR* gene (8). Taking into account that the HER2 receptor predominantly undergoes heterodimerization with EGFR, the narrowing of the binding pocket for ATP, resulting from a mutation in exon 20 of the *EGFR* or *HER2* genes in heterodimer EGFR/HER2, leads to the increased activity of the tyrosine kinases of those receptors (9). This results in an increase of the phosphorylation of further signal proteins, cancer cell proliferation and resistance (or declined susceptibility) to reversible EGFR TKIs (10).

In the future, the presence of the A775YVMA or M774AYVM mutations in the *HER2* gene may be a potential predictive marker of effectiveness of HER family TKIs and be a target for new molecular targeted therapies. The detection of an insertion in exon 20 of the *HER2* gene may play a role in therapy design, and be as important as the current assessment for T790M mutations in the *EGFR* gene, which appears to be a main cause of resistance for reversible EGFR TKIs (50% of all acquired resistance). It should be noted that the brain is the most frequent location for metastases of lung adenocarcinoma. However, there is limited evidence on the prevalence of *HER2* gene mutations in metastatic NSCLC and in patients with histologies other than adenocarcinoma.

## Materials and methods

### Patients

The present study retrospectively analyzed 143 patients (99 male and 44 female) ranging in age between 38 and 81 years (59.8±8.8 years), for whom paraffin-embedded cancer tissue from NSCLC metastatic lesions in the brain was available. The patients underwent routine neurosurgical procedures with a palliative aim. In 32 patients, material from the primary tumor, obtained during thoracoscopy, intrabronchial, transbronchial or transthoracic biopsy, was available. Written informed consent was obtained from all patients. This study was approved by the Ethics Committee of the Medical University of Lublin, Poland (No. KE-0254/131/2011).

Lung adenocarcinoma was diagnosed in 61 patients (42.6%). Squamous and large cell carcinomas were confirmed in 23 (16.1%) and 21 (14.7%) cases, respectively. In 38 patients (26.6%) the NSCLC subtype was impossible to assess and they were diagnosed as not otherwise specified (NOS). The median survival time from lung cancer diagnosis to death was 9.2 months. None of the patients were treateadted with EGFR TKI (EGFR TKI naïve).

### Identification of mutations

DNA was isolated from paraffin-embedded material containing metastatic lesions and from primary tumor tissues using a QIAamp DNA FFPE tissue kit (Qiagen, Valencia, CA, USA). Estimation of the insertion in exon 20 of the *HER2* gene was conducted using a PCR reaction with primers flanking the mutated region of the *HER2* gene. Primers were fluorescently labeled (Cy5). Amplified DNA fragment length analysis (DNA-FLA) was applied using an ALF Express II sequencer (Amersham Pharmacia Biotech AB, Uppsala, Sweden) in polyacrylamide gel. The *HER2* gene mutation was also confirmed by the native electrophoretic separation multi-temperature single-strand conformation polymorphism (MSSCP) technique (11), which allows separation of the different conformers of single-stranded (ss)DNA fragments, in order to differentiate wild-type *HER2* from mutated-type *HER2*. In the MSSCP-based minor variant enrichment procedure, the PCR products were analyzed at strictly controlled temperatures (±0.2°C) using dedicated equipment, the DNAPointer^®^ System (BioVectis, Warsaw, Poland), as described by Kaczanowski *et al*(11). In general, the PCR products were heat denatured and ssDNA conformers were resolved on 9% polyacrylamide gel with 5% glycerol in native conditions (TBE buffer) at three different temperatures (35, 25 and 15°C) during one run. Subsequently, the DNA bands were visualized by silver nitrate staining (SilverStain DNA kit; BioVectis). Fragments of the MSSCP gel containing the bands of interest were cut out and the ssDNA was eluted and re-amplified using primers and PCR conditions as described previously. For subsequent DNA Sanger sequencing (12) a 1/10 volume of obtained PCR products was used (3730xl DNA Analyzer, Applied Biosystems, Carlsbad, CA, USA).

Additionally, the deletion in exon 19, substitution L858R in the *EGFR* gene and mutation V600E in the *BRAF* gene, were assessed in the analyzed material.

## Results

**Figure 1:**
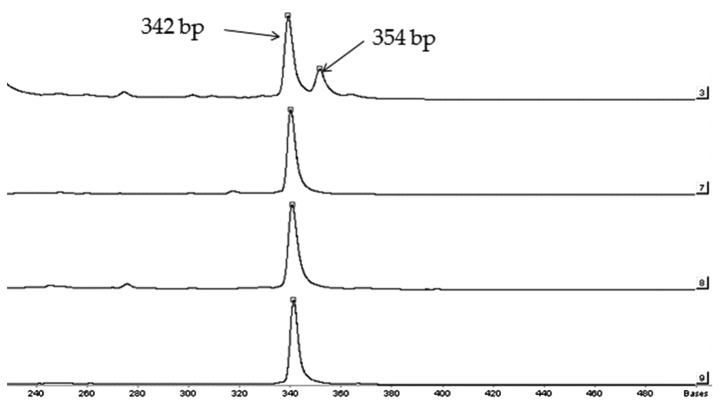
Example of *HER2* gene mutation analysis. The double peaks on path 3 refer to two PCR products, differing in base pair number: The shorter, consisting of 342 base pairs, is typical for the *HER2* wild-type (WT) gene and the longer (insertion of 12 base pairs), is characteristic for mutated (MT) *HER2*. The single peak, containing 342 base pairs on paths 7–9 indicates the presence of only WT *HER2* genes.

**Figure 2:**
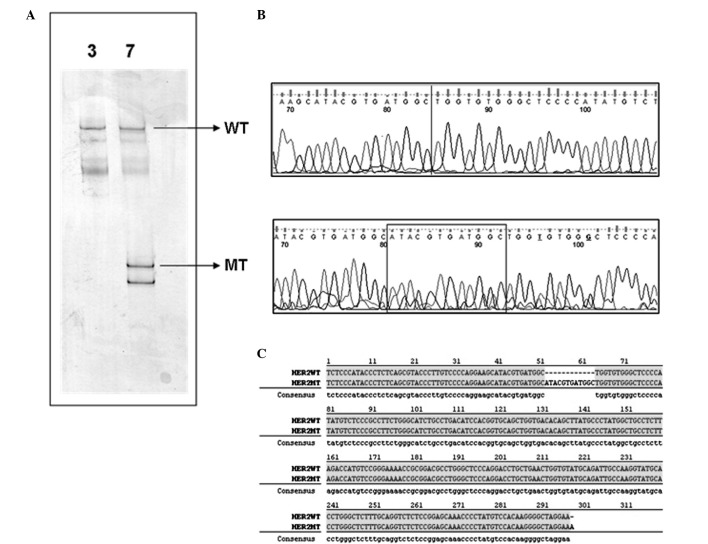
MSSCP electrophoresis easily differentiates between two genetic variants of *HER2*. (A) MSSCP electrophoretic profiles of samples 72 (line 3) and 7 (line 7). PCR products of exon 20 *HER2* gene amplification were heat denatured and ssDNA was separated on 9% polyacrylamide gel using the MSSCP method under optimal electrophoretic conditions. DNA bands were visualized with silver stain. ssDNA bands corresponding to wild-type (WT) and mutated (MT) sequences are indicated by arrows. (B) Partial DNA chromatograms showing the presence of 12 nucleotide insertions in the DNA sequence obtained from the ssDNA band representing mutated *HER2* amplicon (frame). The line in the WT partial DNA chromatogram points to the lack of sequence characteristics for the mutated genetic variant. (C) Sequence alignment of WT and MT *HER2* amplicons detected in sample 7. The 12 nucleotide insertion is visible. MSSCP, multi-temperature single-strand conformation polymorphism; ssDNA, single-stranded DNA.

The insertion in exon 20 of the *HER2* gene (insertion version A775YVMA or M774AYVM) was indicated in a single patient, who only had material from the NSCLC metastatic lesion available (0.67% of all analyzed patients and 1.5% of adenocarcinoma patients; Fig. 1). The mutation was confirmed by the MSSCP technique (Fig. 2). The patient was a 77-year-old non-smoking male, with advanced poorly-differentiated lung adenocarcinoma with metastases in the cerebellum. Due to the low performance status following neurosurgery, and taking into account the advanced stage of the disease, the patient was not treated with chemotherapy, radiotherapy or molecular targeted therapy. Additionally, molecular assessments led to exclusion of other activating mutations in exons 19, 20 (T790M) and 21 (L858R) of the *EGFR* gene, as well as V600E substitution in the *BRAF* gene in the patient with the *HER2* mutation.

In total, 9 activating mutations (6.3%) of the *EGFR* gene were detected in brain samples, these consisted of three delE746-A750 deletions of 15 base pairs in exon 19 (2.1%) and six with L858R substitutions in exon 21 (4.2%). Two deletions in exon 19 were detected in giant cell carcinoma patients and one in an adenocarcinoma patient, while all L858R substitutions were diagnosed in adenocarcinoma patients. Evaluation of the primary tumors revealed *EGFR* mutations similar to those in corresponding metastases: One delE746-A750 deletion in exon 19 and one L858R substitution in exon 21. Additionally, V600E substitutions in the *BRAF* gene were not detected in the brain metastases of the NSCLC patients.

Additionally, the results obtained using PCR analysis were verified with the use of the MSSCP method. As shown in Fig. 2, MSSCP separation of exon 20 *HER2* sample numbers 72 (line 3) and 7 (line 7), recognized previously as the wild-type (WT) and mutated variant (MT), respectively, revealed their distinct electrophoretic profiles. Sample 7 contained additional ssDNA bands, which were not observed in sample 72 (Fig. 2A). It was assumed that similar ssDNA bands detected in each of the two samples indicated the WT *HER2* genetic variant. Considering that the MSSCP method is based on non-denaturing polyacrylamide electrophoresis, additional ssDNA bands suggested the presence of DNA conformers, representing additional amplicon sequences. To verify this hypothesis, indicated ssDNA bands were cut out from the gel and the DNA was recovered, re-amplified and Sanger sequenced. Further analysis of the obtained DNA sequences confirmed our suppositions (Fig. 2B). According to the BLAST database, the ssDNA bands observed in samples 72 and 7, corresponded to the WT *HER2* sequence. Additional ssDNA bands in sample 7 contained the mutated *HER2* sequence with a 12-nucleotide insertion. The described results fully confirmed that sample number 7 was a mixture of two genetic variants of the *HER2* amplicon. The comparison of the WT and the MT DNA sequence detected in sample 7 is illustrated in Fig. 2C.

## Discussion

The present study has shown that primary *HER2* gene mutations are detectable in Caucasian patients with NSCLC. Furthermore, *HER2* gene mutations were indicated in metastatic lesions of lung cancer in the cerebellum, which, to the best of our knowledge is the first report of this worldwide. *HER2* gene mutations are believed to be responsible for the development of these lung cancer types, which occur independent of smoking and are mainly adenocarcinomas. However, the prevalence of *HER2* gene mutations in Caucasian patients is extremely low; in the present study, it has been recorded as occurring in <1% of patients with NSCLC.

Shigematsu *et al* have searched for *HER2* gene mutations in 671 primary NSCLC tumors and 80 NSCLC cell lines, as well as in other types of cancer (14). The authors identified different insertions in exon 20 of the *HER2* gene in 11 patients with NSCLC (1.6%) and in one lung adenocarcinoma cell line (NCI-H1781). This mutation was not observed in other types of cancer, including 55 SCLC tumors. *HER2* gene mutations were more frequent in non-smokers (3.2%; 8/248) and were present solely in patients with adenocarcinomas (2.8%; 11/394). Additionally they were more frequent in female (2.7%; 7/258) compared with male (1%; 4/413) patients. Notably, only one mutation was diagnosed in Caucasian patients (0.7%; 1/137) (5). In another study of 95 patients with NSCLC, Sasaki *et al* described only one non-smoking female patient with lung adenocarcinoma harboring an insertion of 12 nucleotides in exon 20 of the *HER2* gene (6). Buttitta *et al* diagnosed *HER2* gene mutations in 9 out of 403 Caucasian patients with lung adenocarcinoma (2.2%), but only 7 mutations were determined to be an insertion of 12 nucleotide pairs in exon 20. Mutations were more frequent in female (4.1%) and non-smoking (3.1%) patients with bronchioalveolar cancer (6.2%), however, they were also indicated in male patients (1.8%) and smokers (1.9%) (7).

The data from the aforementioned studies, as well as that from the present study, are contrary to the data of Stephen *et al*, which showed that mutations in the tyrosine kinase domain of the *HER2* gene (different *HER2* mutations and abnormalities) are observed in up to 4% of patients with NSCLC (120 primary NSCLC tumors tested), of which, 10% of patients had adenocarcinomas (8). Moreover, Li *et al* described as many as 12 *HER2* gene mutation carriers in a group of 202 (6%) non-smoking Asian patients who underwent surgery for treatment of lung adenocarcinomas (10).

Assuming that *HER2* gene mutations are so-called driver mutations, which drive the epithelial cells into carcinogenesis, and that they do not coexist with other driver mutations (in the present article the coincidence of insertions in exon 20 of the *HER2* gene with EGFR and BRAF gene mutations was not confirmed), patients with these mutations may require specifically targeted therapy against the tyrosine kinase of *HER2*. However, TKIs that inhibit only EGFR tyrosine kinase activity (gefitinib and erlotinib) in cases of only *HER2* gene mutation are ineffective. The reversible, dual EGFR and HER2 tyrosine kinase inhibitor, lapatinib, has showed certain activity in cell lines, but this is not clinically relevant in patients with NSCLC (13,14). The irreversible ErbB family receptor blocker, afatinib (BIBW 2992), which inhibits EGFR, HER2 and HER4 tyrosine kinases, has been shown to be effective in the elimination of cancer cells with *HER2* gene mutations in cell lines and animal models (15–19). Moreover, the first case reports of afatinib effectiveness in female patients with lung adenocarcinoma who are carriers of *HER2* gene mutations have been presented (20). There are ongoing *in vitro* experiments evaluating the feasibility of the use of combined therapy with afatinib and sirolimus (an mTOR inhibitor) in patients with *HER2* gene mutations (4). Moreover, trastuzumab, a monoclonal antibody directed against the extracellular *HER2* domain, which has effectively been used in breast cancer patients with *HER2* overexpression, may be effective in patients with NSCLC that harbor *HER2* gene mutations (21).

Based on the overall data we may conclude that gene profile analysis in cancer patients may extend the scope of molecular therapies used in patients with NSCLC. Moreover, in the near future, the personalized therapy of NSCLC based on the assessment of numerous different gene mutations in cancer cells may become a reality.
